# Multidisciplinary management of patients with cancer in France: The SINPATIC qualitative study

**DOI:** 10.1080/13814788.2024.2380722

**Published:** 2024-07-29

**Authors:** Laura Moscova, Matthieu Lustman, Jacques Cittée, Sébastien Dawidowicz, Florence Canoui-Poitrine, Christophe Tournigand, Kelly Perlaza, William Mirat, Emilie Ferrat

**Affiliations:** a Département de Médecine Générale, Faculté de Médecine, Université Paris-Est Créteil (UPEC), Créteil, France; b Pôle PluriProfessionnel de Santé de Coulommiers, Coulommiers, France; c Pôle de Santé Pluriprofessionnel 94, L'Haÿ-les-Roses, France; d Maison de Santé Universitaire de Sucy-en-Brie, Sucy-en-Brie, France; e Univ Paris Est Creteil, INSERM, IMRB, CEpiA Team, Creteil, France; f AP-HP, Henri-Mondor Hospital, Public Health department, Créteil, France; g APHP, Henri-Mondor Hospital, Oncologie médicale, Université Paris-Est Créteil (UPEC), Créteil, France; h Centre Municipal de Santé Salvador Allende, La Courneuve, France; i Maison de Santé de Torcy, Torcy, France; j Maison de santé Pluriprofessionnelle Universitaire de Saint-Maur-des-Fossés, Saint-Maur-des-Fossés, France

**Keywords:** Cancer, primary care, care coordination, care pathways, qualitative research

## Abstract

**Background:**

Health policymakers have tried to improve the care pathway for cancer patients by improving collaboration between participating healthcare professionals by involving the general practitioner (GP).

**Objective(s):**

To explore how patients, GPs, oncologists and nurses interacted and how they perceived, in their practice, professional roles, collaboration, and cancer care pathways.

**Methods:**

Between January 2018 and December 2021, we conducted a qualitative study that combined phenomenology and a general inductive analysis, based on semi-structured interviews with cancer patients and their GPs, oncologists, and nurses in France.

**Results:**

Our analysis of 59 interviews showed that the stakeholders had different perceptions of the cancer care pathway. Task division was implicit and depended on what each health professional thought he/she should be doing; this led to the blurring of certain tasks (announcement of the diagnosis, coordination, and follow-up). The healthcare professionals were stuck in frameworks centred on their own needs and expectations and were unaware of the other health professionals’ needs and expectations. Outside the hospital, GPs and nurses worked in isolation; they were not aware of the other stakeholders and did not communicate with them. GPs and nurses justified this attitude by the lack of a perceived need. Interprofessional communication varied as a function of the needs, involvement and knowledge of the other health professionals and was often mediated by the patient.

**Conclusion:**

In the cancer management in France, to improve cancer care pathway, there is a need to train healthcare professionals in interprofessional collaboration delivering care tailored to patient needs and preferences.

## KEY MESSAGES

In the management of patients with cancer, the division of tasks between health professionals was not clear and was not discussed by the group.

Communication within the health professionals was mediated often by the patient.

Interprofessional collaboration is strongly encouraged by France’s public health policies but was not mentioned or put into practice by the health professionals.

## Point of Interest

In the management of patients with cancer, the division of tasks between stakeholders was not clear and was not discussed by the group.

Communication within the professionals was mediated often by the patient.

Interprofessional collaboration is strongly encouraged by France’s public health policies but was not mentioned by the stakeholders.

## Background

**Figure 1.**
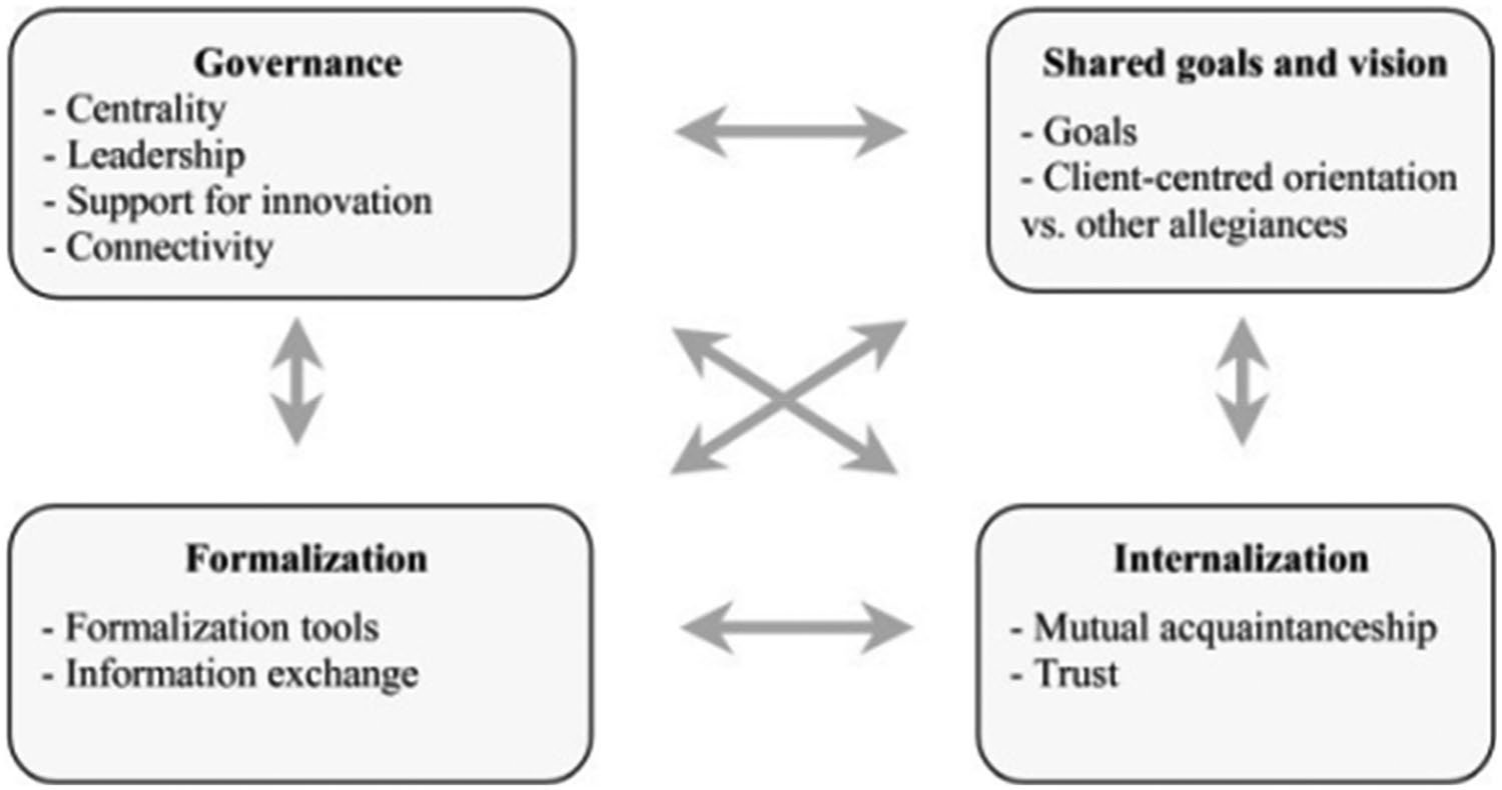
The four-dimensional model of collaboration (from D’Amour et al.).

Healthcare systems in general and primary care organisations in particular are changing in response to demographic and epidemiologic challenges [1,2]. Cancer is a particular challenge: the rising incidence, the complexity of treatment (including orally administered chemotherapy), and the long course of the disease means that general practitioners (GPs) are increasingly involved in cancer care [3,4]. Consequently, patients with cancer are followed by different healthcare professionals (including surgeons, oncologists, primary care physicians, nurses, nutritionists, psychologists, social workers…) [5,6]. This situation may lead to fragmented, poorly coordinated care [6]. With regard to cancer, a number of studies have shown that interprofessional collaboration has an impact on patient satisfaction, quality of life, and continuity of care [7–9]. Sequential, parallel and shared cancer care by GPs and oncologists has been described in the literature [10,11]. In sequential care, cancer patients are followed up by the oncologist only and do not consult their GP until after the cancer has been treated. In parallel care, the patients continue to be treated by their GP for conditions other than cancer. In shared care, cancer patients are followed up by the GP and the oncologist together. However, the GP’s involvement in cancer care varies from one country to another as a function of how the healthcare system is organised [9,12–14]: in countries with gatekeeper healthcare systems (such as The Netherlands or the UK), GPs generally coordinate care and have a longstanding, personal relationship with their patients. In France, specialists take the lead once the patient’s cancer has been diagnosed and treatment has started; primary care teams often lose contact with their patient. Moreover, GPs and oncologists may have different opinions about shared care: although patients and GPs tend to favour greater GP involvement in the follow-up process [15,16], some oncologists disagree because they think GPs lack sufficient skills [17]. Interprofessional collaboration has been defined by D’Amour as a structured group action with a common objective, defined operating procedures, awareness of interdependencies, and regulatory mechanisms [18] (Figure 1). Interprofessional collaboration is a complex and specific process influenced by ideological, organisational, structural and relational factors [19]. With regard to cancer management, the role of health professionals and patients and their responsibilities are not always clearly defined. For example, GPs may not be sufficiently informed about cancer management. Other problems include a lack of communication between healthcare professionals, a feeling of exclusion, and a breakdown in GP care [20]. To date, few studies have investigated the health professionals’ opinions of their expected roles in the management of cancer [17,21]. However, none of the previous studies compared the main health professionals’ opinions and experiences of collaboration and care pathways. Therefore, the objective of the present study was to explore how patients, GPs, oncologists and nurses interacted and how they perceived, in their practice, professional roles, collaboration, and cancer care pathways.

## Methods

### Study design

The French SINPATIC study focused on the interprofessional management of patients with a solid cancer (colorectal, lung, breast or prostate cancer). To meet our research objective, we combined phenomenology with a general inductive analysis. The phenomenological approach was used to explore how interprofessional collaboration, professional roles and care pathways make sense in the health professionals’ practice and in patients’ pathway; we analysed the data drawn from the subjective experiences of each health professional and patient in the pathway [22–24]. We also used a general inductive approach to analyse emerging categories in the collected data, in order to compare them with D’Amour’s reference framework [18,25]. Between January 2018 and December 2021, we conducted semi-structured interviews with cancer patients, GPs, oncologists, nurses and other healthcare professionals in the Ile-de-France region of France. The study’s methods and results were reported in accordance with the Consolidated Criteria for Reporting Qualitative Studies checklist [26].

### Study sample

The sampling was purposive; we sought to include (i) patients who differed in terms of sex, area of residence, tumour site, the time since the multidisciplinary cancer team meeting (more or less than 3 months), the disease stage, the treatment phase (pre-/during/post-), and the treatment intent (curative or palliative), and (ii) healthcare professionals who differed in terms of sex, age, area of activity (rural vs. urban) and, for GPs involvement in the training of students. Eligible GPs, nurses and oncologists were contacted by phone and invited to participate in the study. The patient inclusion criteria were defined by an advisory board, in order to obtain a broad range of participants and points of view. The patients were identified and recruited by their own oncologist or own GP after the latter had joined the study. The study’s advisory board had informed the oncologists and GPs of the patient inclusion criteria before the physicians started to recruit patients. To limit selection bias, both outpatients and hospitalised patients were recruited.

### Data collection

Audiotaped, individual, semi-structured interviews of patients were conducted at home, in hospital, or in their GP’s office. The GPs and nurses were interviewed in their office, and the oncologists were interviewed at their hospital. The interview guides for each health professional and patientwere developed by an advisory board comprising GPs (WM, EF, LM, KP, SD, and JC), sociologists (ML and GP), and oncologists (CT and AL). The guides were refined by the advisory board after the first 12 interviews. The interview guide covered the perception of cancer patients, the perception of professional roles, collaboration, care pathways, and interactions between caregivers (Online supplements S1 to S4). The questions on collaboration were developed on the basis of D’Amour’s model [18].

### Data analysis

All the interviews were transcribed word-for-word and then analysed by six pairs of researchers (EF/LM; WM/ML; SD/KP; KP/ML; ML/LM; LM/GP) [22]. Each researcher worked independently, in order to increase the level of intercoder reliability. The meaningful units in each transcript were identified, grouped into major emergent themes and then discussed by the pairs of researchers. Emergent themes were compared until a consensus was achieved within each pair and then across all the pairs.

The analysis comprised three steps. Firstly, we analysed the experiences of all the patients, GPs, oncologists, and nurses separately. Secondly, we compared the points of view and experiences within each pair, triad or quartette (i.e. an intragroup analysis of the patient vs. his/her GP, oncologist and/or nurse). Lastly, we compared experiences and points of view across groups (i.e. in an intergroup analysis).

**Figure 2.**
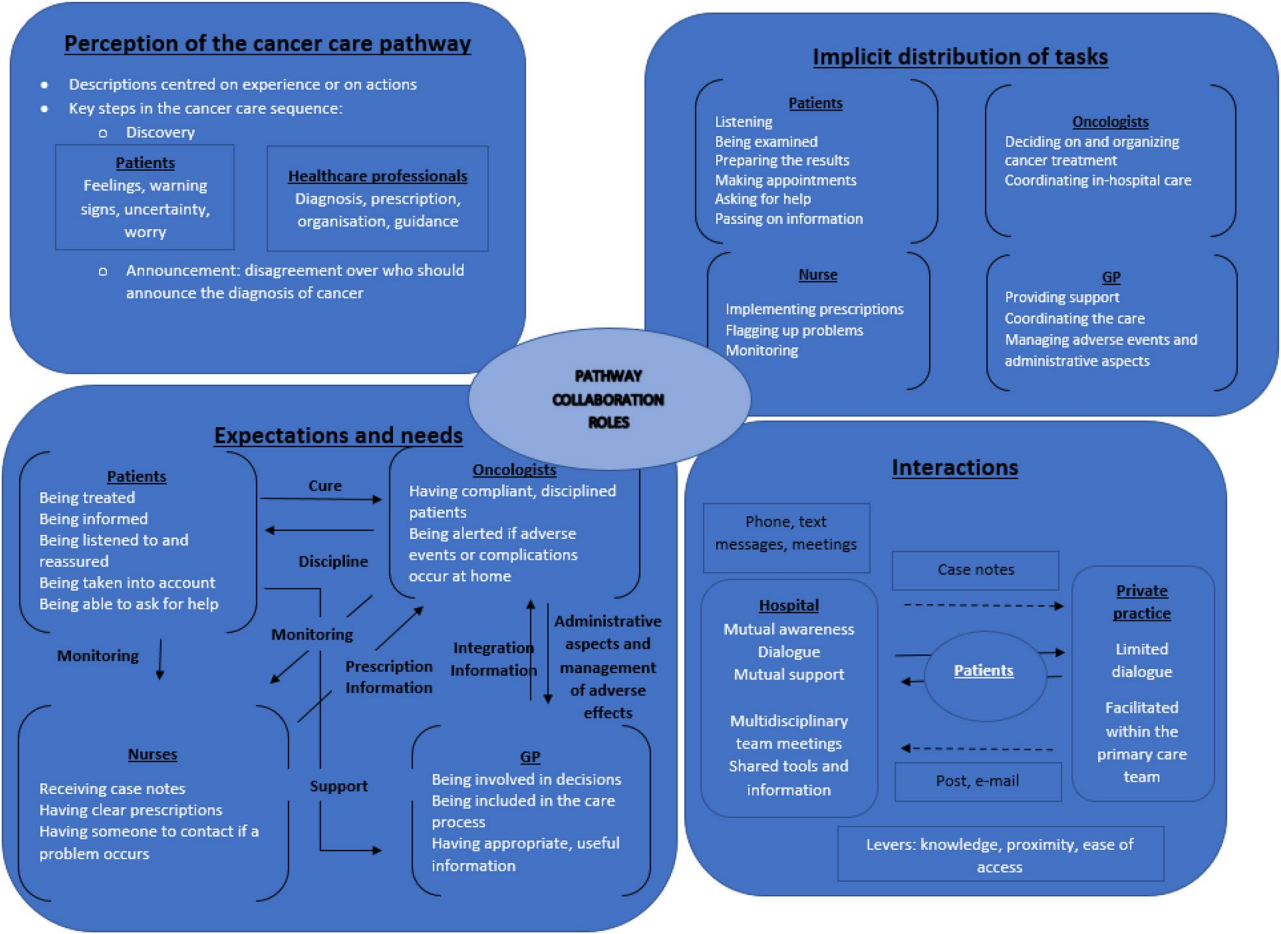
The health professionals and patients’ perceptions of professional roles, collaboration, and care pathways. The direction of solid arrows indicates the burden of the expectation (e.g. the arrow marked “Cure” indicates that the patient expects the oncologist to cure the cancer). The direction of dotted arrows indicates the direction of information transmission (e.g. case notes are sent by the hospital to the GP).

On the basis of this analysis, we graphically summarised the health professionalsand patients’ perceptions of professional roles, collaboration, and care pathways (Figure 2). The figure is intended to illustrate factors that influence perceptions, interactions, and the implementation of interprofessional collaboration in practice.

### Ethics

Participants were informed that their data would be anonymized and gave their consent to participation. The study data were processing in accordance with the guidelines issued by the French National Data Protection Commission (Paris, France; reference: MR003 2104875v0). The SINPATIC study was approved by an independent ethics committee (Paris, France; reference: 01061722).

## Results

Fifty-nine people (19 patients, 17 GPs, 15 oncologists or other specialists, and eight nurses) agreed to be interviewed in the SINPATIC study. The 59 participants formed seven quartettes (i.e. the patient and his/her GP, oncologist and cancer nurse) and - when at least one of the health professionals (the GP, specialist or nurse) was missing - seven triads (six patient-GP-oncologist triads and one patient-GP-nurse triad) and five pairs (two patient-oncologist pairs and three patient-GP pairs).

The health professionals and patient’ characteristics are summarised in Table 1. Patients were aged on average 63 y, *n* = 11/19 patients were female and *n* = 16 were undergoing curative treatment. Among GPS, *n* = 13/17 were teachers and *n* = 15 worked in health centre or group practice. Among oncologists *n* = 11/15 worked in university hospital and *n* = 3/8 nurses had an oncology training.

**Table 1.** Characteristics of the health professionals and patients included in the SINPATIC study.

	**Patients***N* = 19	**General Practitioners***N* = 17	**Medical specialists***N* = 15	**Nurses***N* = 8
**Sexe**				
*Female*	11 (58%)	6 (35%)	11 (73%)	7 (88%)
**Age** (means ; [min-max])	63,3 [36-84]	54,8 [32-76]	48,2 [36-65]	41,5 [37-51]
**Type of cancer**				
*Prostate*	4 (21%)			
*Breast*	9 (47%)			
*Colonorectal*	4 (21%)			
*Lung*	2 (11%)			
**Type of treatment**				
*Curative*	16 (84%)			
*Palliative*	3 (16%)			
**Status of cancer**				
*Local*	11 (58%)			
*Metastatic*	8 (42%)			
**Type of practice**				
*Solo practice*		2 (12%)		
*Group practice*		8 (47%)		
*Health Centre*		7 (41%)		
**Location of practice**				
*Urban*		12 (71%)		
*Rural*		5 (29%)		
**Teaching activity**		13 (76%)		
**University hospital**			11 (73%)	
**Medical specialty**				
*Oncologists*			11 (73%)	
*Gynaecologist*			3 (20%)	
*Surgeon*			1 (7%)	
**Oncology training**				
*Yes*				3 (38%)

Four themes were identified (Figure 2): perceptions of the patient’s medical history and background, the implicit distribution of tasks and a limited coordination, the health professionals and patients’ expectations and needs, and interactions between them. To illustrate each theme, a selection of quotes is shown in Table 2.

**Table 2.** A Selection of verbatim comments from the SINPATIC health professionals and patients.

Themes	Verbatims
*Perceptions of the patient’s medical history and background*	‘And then one day, I had bad bronchitis after the flu and Dr F. examined me carefully. She listened to my back and heart, and then she put her hand there and asked “What’s that?” because it was big.’ *P2* ‘I talked to her again about her screening, and then she admitted out of the blue - at the end of a consultation to renew her prescription for antihypertensives - that she had a lump but didn’t want to tell me about it, because, well, she knew that it was cancer. And so then I asked her if I could examine it. She agreed and, effectively, I felt a lump in her breast’. *GP2* ‘This is a patient who was not at the start of the cancer care pathway. I saw her exactly at the time when signs of metastatic recurrence first appeared’. *O2* *This theme was not mentioned by N2* ‘And it was only then - and this is the strange thing - in October 2016 that someone said the word “cancer” for the first time. It took three years for someone to tell me “you have cancer”. Until then, I had been told “you have a problem, you have ganglions, you have metastases” but the word “cancer” only appeared in October 2016’ *P1* ‘In fact, it was the haematologist. It is not at all usual but as he [the patient] had a first oncological disease, it was the haematologist who told him about the prostate cancer and it was the haematologist who presented the case at his urology team meeting’*O1* ‘I think that I did it [announced the diagnosis] because Mr F trusted me and he kept me informed on a very regular basis. I didn’t need to intervene because there were lots of other physicians involved in the follow-up’ *GP1* *This theme was not mentioned by N1* ‘In March, I started to have stomach pain. I didn’t take it seriously at first, except that it didn’t go away. It lasted two months. […]. I was taken to the emergency department at X Hospital. They found that I had bowel obstruction and that I had to have surgery on the following day. So that went OK, I had the operation, and so I spent two weeks in hospital.’ *P11* ‘The diagnosis, it wasn’t me who announced it.’ *GP11* ‘I think that she had been operated on in another hospital and they asked me for an opinion. Well, I don’t remember which hospital it was. They asked me for an opinion on social, um, medical management. She came for a consultation, and I think that, ultimately, she wanted to be treated at X.’ *O11* *This theme was not mentioned by N11*
*Failure to discuss tasks distribution and coordination within the hospital alone*	‘[Interviewer]: How do they work together? [P3]: Well, I don’t know at all about that…. I don’t know’ *P3* ‘I coordinate. I need to coordinate, otherwise I don’t feel that I’m in my role as a GP. So for Mrs L, it’s the same – I am the physician who does it all. In as much as I say “I do it all” *GP3* ‘In fact, all this organisation happens through weekly or fortnightly concertation meetings where all the stakeholders meet. It’s that that helps, the meetings that are obligatory. It’s that that helps’ *O3* ‘Well, sometimes, what’s strange can be the lack of agreement between the hospital physicians and the private practitioners’ *N3* ‘They [the oncologists] prescribed drugs to prevent the nausea – which didn’t prevent anything, in fact – and then in case I caught a cold or anything, they prescribed antibiotics for me because if I had fever, it could be dangerous. But they were telling me that I still had to go and see a doctor before taking them.’ *P4* ‘She comes to see her GP so that I can extend her sick leave. It’s a good reason too because she was working.’ *GP4* ‘Most of my patients don’t see their GP any more. The patients say “no, I don’t go to see him/her [the GP] because he/she says that it’s the oncologist that you need”, and you are obliged to renew prescriptions that aren’t even yours’ *O4* *No nurse in this quartette*
*The stakeholders’ expectations and perceived needs*	‘I reckon that the GP is the one who has to be aware of all your major or minor health niggles. I am being treated very well at the hospital, and so I don’t know what more the GP can do… It is not with him that the disease started.’ *P11* ‘Patients with high blood pressure or diabetes, that’s my job. Diagnosing cancer, that’s my job. Treating it and following it up, that’s not my job, or at least it’s not my core activity.’ *GP11* ‘I expect that if my patient has a temperature of 38.5° or a sore throat, or pain urinating or pain somewhere, then they should see the GP and not me. So, in my view, I think that the GP is useful - very useful at the start of treatment to set up the administrative procedure for the full reimbursement of healthcare costs by the social security, that’s clear! And then for the treatments, managing all the small everyday problems that don’t necessarily require action from the oncologist’ *O11* ‘I say that the patients only go to see the oncologist, but we’re the same – we’re only going to refer people to the oncologist. Even if we have a general problem, we refer them straight away to the oncologist.’ *N11* ‘And I find that oncologists and physicians in general do not know the patient, they never take account of the patient’s wish to be kept informed or their ability to analyse things too. And I want to be given data so that I can understand, analyse and decide.’ *P2* ‘I didn’t have any records for the patient on chemotherapy – nothing. I didn’t get any advice on how to manage the side effects.’ *GP2* ‘The main stakeholder in patient management is the oncologist […] I have lots of patients who are far away, even in physical terms, and that there’s all that management of side effects to do, and it’s good to have someone (the GP) who can help me manage that.’ *O2* ‘And yes, it’s true that this is a big problem in private practice because sometimes, if we don’t dig a bit and we don’t ask a few questions, we turn up and the person says “I have these injections” but they don’t tell us why. So, we have to ask questions and it’s true that we rarely get a follow-up file from the hospital or perhaps they don’t give it to us – I don’t know.’ *N2*
*Interactions between the stakeholders*	‘In principal, he gets some reports. I know that my urologist has sent him one, and the pulmonologist was supposed to – I hope that she did, although I don’t know if there is any dialogue between them.’ P6 ‘[Interviewer]: And so what relationships do you have? [GP6]: There are no relationships. A report from time to time, that’s all. I read the letter, of course, but then they don’t say much in the letter.” GP6 ‘Knowing the teams and the various people involved well, that helps to circulate information more quickly, in fact. For the patients, it’s enormously reassuring for them to know that the oncologist, the radiotherapist, and the anaesthetist know each other. We can exchange information very easily, which is very reassuring for the patient’ O6 *No nurse in this quartette* ‘But one day, she tried to get the oncologist (Dr V) on the phone. She didn’t get through, so she called her back. I think that Dr V wants to communicate too, it’s someone who’s at the hospital and wants to communicate with my GP.’ *P2* ‘The only one I’ve been able to contact is Mrs V. We communicate by e-mail. She was very nice. I called her once or twice when he began to have metastases but it’s true that she’s the only one I’ve been able to form a link with. The others were not available. I didn’t have the oncology records. I received discharge letters but not at the right time.’ *GP2* ‘I talked to her GP regularly at first (and less often now less). I referred her to a palliative care network and I had a good relationship with the team who looked after her.’ *O2* ‘And yes, it’s true that this is a big problem in private practice because sometimes, if we don’t dig a bit and we don’t ask a few questions, we turn up and the person says “I have these injections” but they don’t tell us why. So, we have to ask questions and it’s true that we rarely get a follow-up file from the hospital.’ *N2*

P, patient; N, nurse; GP, general practitioner; O, oncologist or other cancer specialist.

### Perceptions of the patient’s medical history and background

The patients talked mainly about how their cancer was discovered, the role of their family and friends, and their experience of the disease, whereas the healthcare professionals summarised the patient’s background and life in biomedical terms. The patients were especially marked by the cancer discovery step, which was lengthy, complex, and worrying. However, the patients were actively involved in this step by flagging up a symptom or an unusual event, making appointments, and waiting until the diagnosis was announced by GPs or specialists.

The oncologists summarised the patient’s life in technical terms, with a focus on the cancer. A few oncologists considered the patient’s family and friends and experience of the disease and adjusted the treatment accordingly. The oncologists and cancer nurses did not talk much about the discovery because they were not involved in this step.

The nurses often lacked information about the patient’s background and life; their perception of the care pathway was limited and was influenced by what the patients told them.

If they had been involved, the GPs talked about how the cancer had been discovered. Some also commented on the patient’s social environment, the patient-GP relationship, and patient’s experience.

In some quartettes, triads or pairs, we observed that the patients, GPs and/or oncologists disagreed about who had announced the cancer diagnosis. The announcement of diagnosis was described as being fragmented, with no concertation between the oncologist and the GP. The nurses were not usually involved in care at this stage.

### The implicit distribution of tasks and a limited coordination

The roles were described in terms of the tasks that the health professionals had attributed to themselves. Some patients listened to the oncologist and tried to facilitate their work by showing their trust, agreeing to examinations, preparing the results, and making appointments. Others asked for help from their cancer nurse or their GP (in order to gain a better understanding of their medical situation) or from their friends and family (for social issues). They asked questions and sometimes objected the medical decisions, so that their opinion would be taken into account or so that they could participate in their own way. The oncologists described themselves as being responsible for the cancer follow-up: they took decisions, organised the treatment, and coordinated the hospital-based care. Some oncologists considered themselves to be the “physician-in-chief for cancer”. The nurses executed the physicians’ prescriptions and had a technical role. They contributed to patient monitoring and saw themselves as “sentinels” who flagged up problems early. The GPs provided patients with psychological support, managed adverse events and administrative requests, and coordinated care as a whole. The nurses and some of the oncologists thought that the GP was not sufficiently competent for following up cancer patients. Whereas some tasks were shared out implicitly, others overlapped (diagnosis, announcement of cancer, management of adverse events, coordination, and follow-up) and were more difficult to share.

Treatment decisions were made in multidisciplinary team meetings in hospital. The patients, GPs and nurses were well aware of these multidisciplinary team decisions but they didn’t participate, which were integrated into the oncologists’ usual practice. The patients described these treatment decisions as a group consensus made by specialists. This decision was perceived differently from one patient to another: some trusted the specialists’ decision, whereas others negotiated or objected initially before being convinced (usually by the oncologist). Most of the GPs did not contribute to these decisions and did not discuss them. A few discussed the planned care with their patients without consulting the oncologists and had therefore changed the decisions and the treatments. Only five GPs would have liked to have been involved in the decision-making; the others did not express an opinion because they did not feel competent or legitimate.

The oncologists perceived their work as being coordinated because the follow-up was shared with other specialists. Outside the hospital, most GPs and nurses worked alone and did not feel the need to talk to or get to know the other healthcare professionals. Some of the GPs were committed to following up their patients, whereas others had given up because they felt that had not been given that role by the patient and/or the oncologist or felt that it was not their role. Most of the nurses executed the prescriptions and worked without collaborating with either the oncologists or the GPs; their primary partner was the patient.

The health professionals talked about “coordination”, rather than “collaboration”. Coordination appeared to be limited to within the hospital but was sometimes shared with the GP - depending on the role that he had given himself and the space given to him by the patient and/or the hospital staff.

#### The health professionals and patients’ expectations and perceived needs

The health professionals and patients’ expectations differed and there was little coordination between healthcare professionals in this respect. The patients mainly expected to be treated, given information, listened to, reassured, and taken into account. They expected the oncologists to cure the cancer and sometimes wanted to spend more time talking about treatments and adverse events. The patients expected the GP to manage adverse events, listen to them, reassure them, and help with administrative matters (social security and sick leave). Thus, the patients attributed an active, therapeutic role to the oncologist, a support role to the GP, and a technical monitoring role to the nurses.

The oncologists expected (i) the patients to be compliant, (ii) the GPs to manage out-of-hospital adverse events, the end of life at home, and administrative tasks, and (iii) the nurses to monitor the patients at home and to flag up any problems. Furthermore, the oncologists wanted the GPs to commit to the management and make their involvement known but also thought that cancer care was too complex for GPs or that GPs did not wish to be involved.

The nurses thought that the oncologist was the primary stakeholder in cancer care. They expected the oncologist to brief them on the patient and give clear and comprehensive prescriptions. The nurses’ primary care partner was the patient. The nurses expected the patient to give her as much information as possible so that she could understand the situation. The nurses wanted to be able to contact the hospital easily in the event of a problem. They often did not perceive the GP to have a role or utility in the patient’s care.

Some GPs wanted to be more integrated into the care pathway by the hospital team, with involvement in treatment decisions and the provision of patient-specific information of value in the management of adverse events. The GPs also complained that their opinions were not sufficiently taken into account.

#### Interactions between the health professionals and patients

Outside the hospital, dialogue between the healthcare professionals was limited. The oncologists communicated with the other professionals within the hospital by phone, *via* the medical records, or during meetings. Communication between hospital-based professionals and GPs was essentially limited to sending case notes and discharge letters. Most of the oncologists did not initiate communication (other than by sending regular case notes, which were often perceived to be received late by the GPs) and did not express the need to initiate communication with GPs. In most cases, the oncologists did not know who the patient’s GP was. Some GPs took the initiative of contacting the oncologist by phone or by e-mail, whereas other gave up after having difficulty getting in touch with the oncologist. Likewise, the nurses had also little direct contact with the oncologists and complained about not having any information on the patient’s medical status other than prescriptions. In fact, the communication within the quartette was mediated often by the patient, who often served as an intermediary or messenger between the professionals. Several communication tools were used: prescription, patient, case notes, discharge letters, e-mail, phone calls, and phone text messages. The use of these various tools depended on how close to each other the health professionals felt.

It took time and commitment to build awareness of and relationships between the health professionals and patients. The GPs put their energy into their relationship with the patient. Some oncologists also committed themselves to the patient-physician relationship by adjusting the care pathway if the patient was adherent and facilitated the treatment. Lastly, the nurses described a close relationship with the patient through their regular or even daily visits, which enabled them to understand the patients’ feelings. Collaboration was not therefore mentioned spontaneously in the stakeholders’ narrative and thoughts about how to work together were focused on their relationships and, more precisely, on communication that was easy to understand, analyse and comment on.

## Discussion

### Main findings

The patients stated that their cancer pathway was a long process, marked by significant milestones and scattered with moments of uncertainty, doubt, and hope. The announcement was variously described as being expected, prepared for, or sudden. Although the patients expected the oncologist to announce the diagnosis, the other stakeholders did not all agree on this point. Moreover, the same pathway was thus reported differently by the health professionals: (i) an overall narrative by the patients, (ii) a fragmented, “step-by-step” narrative focused on their involvement by the GPs and the oncologists, and (iii) a “closeness” narrative focused on relationships and care by the nurses. Each healthcare professional’s role and tasks were conceived in an isolated, profession-specific manner, with no attention paid to the multidisciplinary nature of care; this appeared to confuse or blur the perception of certain tasks (the announcement, coordination, and follow-up). The work was described as being coordinated by the oncologist, who shared the patient’s follow-up and care with other professionals (e.g. the surgeon and the nurse). Outside the hospital, the GPs and nurses worked in isolation; they were not aware of the other health professionals and did not communicate with them. The GPs and nurses justified this attitude by the lack of a perceived need. Some GPs committed to following up the patient in their own way and claimed roles in pain management, administrative work, psychological support, and a more general coordination role (i.e. less focused centred on cancer). The nurses applied the prescriptions, monitored the patients, and alerted the physicians if necessary; they considered themselves to be technicians or “sentinels”.

“Collaboration” was not mentioned and was mainly described in terms of communication and coordination. The ways of working together were analysed by the patient and the healthcare professional in terms of their relationships and, more precisely, communication. Communication between healthcare professionals was varied according to needs, commitment, and mutual awareness; it was often mediated by the patient, whose role as an intermediary or messenger influenced the interactions between the health professionals.

### Strengths and limitations

To the best of our knowledge, the health professionals and patients’ perceptions and experiences of collaboration, roles and care pathways for given cancer patient have not previously been compared. None of the published studies looked at the group dynamics and interactions. To this end, we conducted individual, semi-structured interviews in order to compare and contrast opinions about the same pathway and to understand the interactions between the health professionals and patients involved in following up the same patient. Other strengths of the present study included the variety of the investigators’ specialties (GPs, oncologists, nurses and sociologists), the purposive sampling, the multiple coding procedures, and the diverse patient sample (with regard to the type of cancer, the treatment intent, the treatment phase and the treatment setting)[26].

Our approach combining phenomenology and general inductive analysis permitted to understand and share, from the clarifying of their practice, the point of view and experience of GPs, nurses and oncologists involved in the care of patients with cancer.

However, we were not always been able to interview full quartettes; some patients did not have a cancer nurse, and some GPs, oncologists and nurses did not reply to our request for an interview.

The fact that none of the patients had free state health insurance (a status that often reflects a low socio-economic level) may have affects our findings. Moreover, half of the GPs in our sample supervised trainee GPs, and many of the oncologists worked in a university hospital; these aspects might have led the physicians to be more aware of the concept of collaboration. Hence, our sample of physicians might not have been representative of interactions in the French health system more widely and so might limit the ability to extrapolate our present results.

The interview guides were based on D’Amour’s model; the latter conditioned our questions and thus constituted a limitation on our inductive approach. However, the interview guide was sufficiently open-ended, and the questions were designed to gain a good understanding of the participants’ experiences [18,22,27].

### Comparison with the literature data

The care pathway for patients with cancer is complex, and the various health professionals’ points of view are not always concordant. Disagreements between oncologists, nurses, GPs and patients about their respective roles in cancer care make it difficult to share tasks and clarify roles [16,17,20,21,28,29].

We found that within a given quartette, the patient and the healthcare professionals often had different expectations; this sometimes led to misunderstandings, criticism, or even conflict. The definition of a common objective appeared to be difficult because each health professional’s actions depended mainly on their needs and professional interests, which were sometimes contradictory [18].

As in the literature, our results showed that the health professionals were not necessarily all involved in the follow-up of cancer patients and that the follow-up for a given patient was not always shared between the stakeholders [10,20]. Other than in multidisciplinary team meetings, healthcare professionals have few opportunities to get to know each other and to understand how the others work. Three types of collaboration can be defined: (i) collaboration in inertia, with leadership battles, no relationships, no negotiation, and no shared responsibility; (ii) collaboration under construction, in which negotiation processes are present and responsibility sharing is fragile; and (iii) collaboration in action (the highest level of collaboration), in which responsibilities are shared, consensuses are formalises, and care is based on continuity and efficiency [30]. In our study, we observed a lack of mutual understanding of other health professionals’ needs, a lack of negotiation over roles and task-sharing, and an absence of conceptualisation and thoughts about collaboration and its common goal. We consider that this corresponded to collaboration in inertia (led by the hospital) and some elements of collaboration under construction within the hospital. In our study and others in the literature, a number of factors may account for collaboration in inertia: the variable level of the GP’s involvement in care, the role in follow-up given to the GP by the other health professionals (i.e. the patient, the oncologist and the nurse), poor awareness among oncologists of the GP’s level of knowledge and skills, and organisational differences between private practices and hospitals [30,31]. Even when GPs wanted to “get involved” more, involvement was limited by the oncologists’ failure to provide information at the right time, and a lack of knowledge, legitimacy and skills [7,21,32,33].

### Implications for practice

In response to the complex challenges faced by cancer patients, interprofessional collaboration has been promoted by healthcare institutions. While a few studies have highlighted the impact of interprofessional collaboration on patient satisfaction, quality of life, and continuity of care [7,9], this is not perceived by the health professionals and does not appear to be a true goal. Interprofessional collaboration requires (i) definition of the rules for task division, (ii) formalised objectives for shared management, and (iii) a sense of integration into a team by getting to know the other members, learning to trust them, and sharing professional skills [34]. To achieve this, the health professionals must develop a formalised care team around the patient, define communication rules and tools, clarify roles, and develop the skills required for interprofessional collaboration [30].

## Conclusion

In routine cancer care in France, the patient, the oncologist, the GP and the nurse do not collaborate. Coordination between the stakeholders is limited; the level of mutual awareness is low, and there is no consensus on the various roles. As a result, collaboration is an ideal promoted by the French institutions in order to improve the cancer care pathway.

To improve cancer care pathway, there is a need to train healthcare professionals in France in interprofessional collaboration delivering care tailored to patient needs and preferences. Experiences in other countries could be helpful to improve management of patients with cancer.

## Supplementary Material

Supplemental Material

Supplemental Material
