# Supplementary material for: Multidisciplinary management of patients with cancer in France: The SINPATIC qualitative study
Source: Eur J Gen Pract. 2024 Jul 29;30(1):2380722. doi: 10.1080/13814788.2024.2380722 (PMC11288201; doi:10.1080/13814788.2024.2380722)
Supplement: Supplemental Material [file IGEN_A_2380722_SM4023.docx]

**Online Supplements. Tables S1 to S4 – Health professionals and patients interview guides**

**S1 – The patient interview guide**

**Theme 1: the cancer care pathway**

- **Can you describe your cancer care pathway, from the first examination up until today?**

Prompts:

-How was the diagnosis made? By whom?

-How is your follow-up organized?

-What are the treatment objectives, in your opinion?

-Which stakeholders have had a role in the diagnosis and follow-up? Which roles?

-Can you tell me about your experience with these various stakeholders?

-Can you tell about any clinical situations that required precise or particular management?

-What could have been or could be improved in your cancer treatment?

**Theme 2: interprofessional collaboration**

- **Can you describe the collaboration between healthcare professionals in your treatment and follow-up? How does the collaboration operate in practice?**

Prompts:

-Can you describe the various healthcare professionals involved in your follow-up and the relationships between them?

-Are there agreements between them, with precise rules?

-Do you think that they communicate with each other? If so, how?

- If there are disagreements between them, how are they resolved?

-How should responsibilities and decisions be shared, in your opinion?

-Do you think that it is important for healthcare professionals to collaborate and exchange information?

-What, in your opinion, does or would facilitate relationships between the healthcare professionals? Collaboration?

-What restricts relationships between the healthcare professionals? Collaboration?

-How could this be improved?

-Overall, how would you define the type of care that you received? (sequential, parallel or shared)

- **Can you tell me about the perceived effects of collaboration on your follow-up?**

Prompts:

-Do you think that collaboration improves your follow-up/treatment? If so, how and why?

**Theme 3: The stakeholders’ respective roles in the follow-up**

- The perceived role of the GP

- **Can-you tell me about your GP and their professional role?**

Prompts:

-What is your GP’s role with regard to your follow-up?

-Can you describe their involvement since the beginning?

-What do you expect from him/her?

-Would you like their role and involvement to be different?

-What could you suggest to improve the situation?

-What is the GP’s role within the specialist medical team with regard to treatment decisions and follow-up?

-What role should the GP have, in your opinion?

-What are the GP’s strengths with regard to your follow-up?

-What are the GP’s weak points/limitations with regard to your follow-up?

-In your opinion, how does (do) the specialist(s) view the GP’s role?

-The perceived role of the oncologist:

- **Can you tell me about the oncologist’s role in your treatment and follow-up?**

Prompts:

-What is his/her role with regard to your follow-up?

-Can you describe its involvement since the beginning of the treatment?

-Would you like his/her role to be different?

-What could you suggest to improve the situation?

-What role should he/she take, in your opinion?

-What are the oncologist’s strengths with regard to your follow-up?

-What are the oncologist’s weaknesses/limitations with regard to your follow-up?

-Did the oncologist ask for the name of your GP?

-Is your oncologist keen to transmit information?

-The perceived role of other healthcare professionals (physicians and nurses)

- **Can you tell me about the roles of the other healthcare professionals involved in your treatment and follow-up?**

Prompts:

-What are their roles with regard to your follow-up?

-Can you describe their involvement since the start of the treatment?

-Would you like their roles to be different?

-What could you suggest to improve the situation?

-What role should they take, in your opinion?

-What are their strengths with regard to your follow-up?

-What are their weaknesses/limitations with regard to your follow-up?

-Did they ask for the name of your GP?

-Were they keen to transmit information?

-The patient’s role in the follow-up:

- **Can you describe your role in the follow-up/treatment of your cancer?**

Prompts:

-What is your role in your follow-up/treatment?

-Could you describe your involvement in the collaboration (if any) between the various healthcare professionals?

-Do you contribute to the transmission of information between them? If so, how?

-In your opinion, which important items of information about your health must be known to the healthcare professionals? Who transmits this information and how?

**S2 – The general practitioner interview guide**

**Could you tell me about you and your medical activity?**

**Theme 1: the cancer care pathway**

How many adult cancer patients are you following up? (the interview is centred on patient X)

We are going to talk about Mr/Mrs X.

- **Can you describe the patient’s care pathway, from the first examination up until today?**

Prompts:

-Can you describe the patient’s medical, psychological and social status?

-How was Mr/Mrs X’s cancer diagnosed?

-What are the treatment objectives for this patient?

-Can you describe any clinical situations that required specific/particular treatment?

-What could have been or could be improved with regard to the treatment?

Is this patient similar to the other cancer patients that you follow up?

**Theme 2: interprofessional collaboration**

- **In your opinion, how did you get on with the oncologist and/or the other healthcare professionals involved in the management of your patient?**

Prompts:

-Can you tell me about the various healthcare professionals with whom you work

-Can you tell me about the resources that are used by the healthcare professionals to work together/ communicate?

- How do you describe your relationships with the other healthcare professionals ? (general atmosphere and trust)

- What, in your opinion, facilitates the relationships?

- What limits the relationships?

- How did trust develop between you?

- How were decisions and responsibilities concerning follow-up shared?

- How do you manage conflicts?

- How could this it be improved?

-Overall, how would you define the type of care that the patient received? (sequential, parallel or shared)**

- **Do you think that dialogue with the other healthcare professionals changes your patient’s treatment?**
- **In your opinion, what would be the ideal type of collaboration for following up cancer patients?**

**Theme 3: The stakeholders’ respective roles in the follow-up**

-The perceived role of the GP

- **Can you tell me about your role/involvement in the treatment of this patient?**

Prompts:

-How would you wish to be involved in the treatment?

-What is your role within the team of specialists concerning treatment and follow-up decisions?

-In your opinion, what are the GP’s strengths with regard to the follow-up?

-What are the GP limitations with regard to the follow-up?

-Is this patient being followed up in the same way as your other patients?

-The perceived role of the oncologist:

- **Can you tell me about the oncologist’s role in the treatment and the follow-up of your patient?**

Prompts:

-How would you wish the oncologist to be involved in the treatment?

-What are the oncologist’s strengths with regard to the follow-up?

-What are the oncologist’s limitations with regard to the follow-up?

-Role of the other healthcare professionals (physicians such as geriatric oncologists and organ specialists, and nurses)

- **Can you tell me about the role of the other healthcare professionals involved in your patient’s the treatment and follow-up?**

Prompts:

-Can you describe their involvement since the beginning of the treatment?

-How would you wish them to be involved in the treatment?

-What are their strengths with regard to the follow-up?

-What are their limitations with regard to the follow-up?

-Role of the patient in the follow-up:

**In your opinion, what would be the patient’s role in the treatment of their cancer?**

Prompts:

- How does the patient contribute to dialogue between you and the other healthcare professionals?

- Do you think that the patient has a role to play in his/her own follow-up? And in the dialogue between healthcare professionals?

**S3 – The oncologist interview guide**

**Theme 1: the cancer care pathway**

- **Can you describe the patient’s care pathway, from the first examination up until today?**

Prompts:

-Can you describe the patient’s medical, psychological and social status?

-How was Mr/Mrs X’s cancer diagnosed? By whom?

-What are the objectives of the treatment for this patient?

-Can you describe the main clinical situations that required specific treatment?

-How is the follow-up organized? Which healthcare professionals have been called on, and how was that done?

-Which healthcare professionals had a role in the diagnosis and follow-up? How was that done?

-What could have been or could be improved with regard to the treatment?

-Is this patient similar to the other cancer patients that you are monitoring?

**Theme 2: interprofessional collaboration**

- **Can you tell me about the healthcare professionals’ collaboration with regard to your patient’s follow-up? How did that happen in practice?**

Prompts:

-What is interprofessional collaboration, in your opinion?

-Can you tell me about the various healthcare professionals with whom you collaborate?

-Can you describe the framework (whether formal or not) that you used to facilitate collaboration? (agreement, protocol, information exchange)

-Can you tell me about the resources that are used by the healthcare professionals to implement this collaboration in practice?

- How would you describe your relationships with the other healthcare professionals? (general atmosphere and trust)

-What, in your opinion, facilitates these relationships?

-What limits these relationships?

-How did trust develop between you?

- How were decisions and responsibilities concerning follow-up shared?

- How do you manage conflicts?

-Do you think that you have the same vision of collaboration as the other healthcare professionals?

-How could this be improved?

-Does the current organisation favour the development of collaboration between healthcare professionals?

-Overall, how would you define the type of care that the patient received? (sequential, parallel or shared).

- **Can you tell me about the practical effects of collaboration on your patient’s follow-up?**

Prompts:

-Do you think that this improves patient management?

-If so, how?

-How does that improve the care pathway for the patient?

-How does that improve the care pathway change for you?

- **In your opinion, what would be the ideal type of collaboration?**

**Theme 3: The stakeholders’ respective roles in the follow-up**

-The perceived role of the GP

- **Can you tell me about the GP’s role in the patient’s treatment?**

Prompts:

-In your opinion, what is the GP’s role in Mr/Mrs X’s follow-up?

-Can you describe the GP’s involvement since the beginning?

-What do you expect from him/her?

-How could his/her role be different?

-What is the GP’s role within the team of specialists? And in treatment/follow-up decisions?

-What are the GP’s strengths with regard to the follow-up?

-What are the GP’s limitations with regard to the follow-up?

-The perceived role of the specialist:

- **Can you tell me about your role in the patient’s treatment?**

Prompts:

-What is your role in your patient’s follow-up?

-Can you describe your involvement since the beginning of the treatment?

-How would you wish to be involved in the treatment?

-What is your role within the team of specialists? And in treatment/follow-up decisions?

-What are the oncologist’s strengths with regard to the follow-up?

-What are the oncologist’s limitations with regard to the follow-up?

-Role of the other healthcare professionals:

-What is their role in your patient’s follow-up?

-Can you describe their involvement since the beginning of the treatment?

-Would you want their role to be different?

-How would you wish them to be involved in the treatment?

-What is your perception of their role within the team of specialists? And in the decisions concerning the treatment/follow-up?

-What are their strengths with regard to the follow-up?

-What are their limitations with regard to the follow-up?

-Role of the patient in the follow-up:

- **Can you describe the patient’s role in his/her follow-up?**

Prompts:

-What is his/her role in the follow-up?

-How does he/she participate in interprofessional collaboration and the transmission of information between healthcare professionals?

- Do you think that the patient has a role to play in his/her follow-up? If so, which roles and how? And why is that?

- Do you think that the patient has a role to play in interprofessional collaboration?

**S4 – The nurse interview guide**

**Theme 1: the cancer care pathway**

- **Can you describe the patient’s care pathway, from the first examination up until today?**

Prompts:

-Can you describe the patient’s medical, psychological and social status?

-How was Mr/Mrs X’s cancer diagnosed? By whom?

-What are the treatment objectives for this patient?

-Can you describe any clinical situations that required specific/particular treatment?

-How was the follow-up organized? Which healthcare professionals were involved and how?

-Which other healthcare professionals could have had a role in the diagnosis and follow-up? How would that happen?

-What could have been or could be improved with regard to the treatment?

- Is this patient similar to the other cancer patients that you are monitoring?

**Theme 2: interprofessional collaboration**

- **Can you tell me about the healthcare professionals’ collaboration with regard to your patient’s follow-up? How did that happen in practice?**

Prompts:

-What is interprofessional collaboration, in your opinion?

-Can you tell me about the various healthcare professionals with whom you collaborate?

-Can you describe the framework (whether formal or not) that you used to facilitate collaboration? (agreement, protocol, information exchange)

-Can you tell me about the resources that are used by the healthcare professionals to implement this collaboration in practice?

-How would you describe your relationships with the other healthcare professionals? (general atmosphere and trust)

-What, in your opinion, facilitates the relationships?

-What limits the relationships?

-How did trust develop between you?

-How se fait the sharing of the decisions and of the responsibilities concerning the follow-up?

-Of quelle façon do you manage your conflicts?

-Do you have the impression of having the same vision that the other healthcare professionals of the collaboration?

-How could this be improved?

-Does the current organisation favour the development of collaboration between healthcare professionals?

-Overall, how would you define the type of collaboration? (sequential, parallel or shared).

- **Can you tell me about the practical effects of collaboration on your patient’s follow-up?**

Prompts:

-Do you think that this improves the treatment?

-If so, how?

-How does that improve the care pathway for the patient?

-How does that improve the care pathway change for you?

- **In your opinion, what would be the ideal type of collaboration?**

**Theme 3: The stakeholders’ respective roles in the follow-up**

-The perceived role of the GP

- **Can you tell me about the GP’s role in the treatment of this patient?**

Prompts:

-In your opinion, what is the GP’s role in Mr/Mrs X’s follow-up?

-Can you describe the GP’s involvement since the beginning?

-What do you expect from him/her?

-Would you like his/her role to be different? If so, how?

-What is the GP’s role within the team of specialists with regard to treatment/follow-up decisions?

-What are the GP’s strengths with regard to the follow-up?

-What are the GP’s limitations with regard to the follow-up?

-The perceived role of the oncologist:

- **Can you tell me about the oncologist’s role in your patient’s treatment and follow-up?**

Prompts:

-What is your/his/her role in the patient’s follow-up?

-Can you describe your/his/her involvement since the beginning of the treatment?

-How would you wish (or wish him/her) to be involved in the treatment?

- What is your/his/her role within the team of specialists with regard to treatment/follow-up decisions?

-What are your/his/her strengths with regard to the follow-up?

-What are your/his/her limitations with regard to the follow-up?

-Roles of the other healthcare professionals:

- **Can you tell me about their roles in your patient’s treatment and follow-up?**

Prompts:

-What is your/their role in your patient’s follow-up?

-Can you describe your/their involvement since the beginning of the treatment?

-How would you wish them to be involved in the treatment?

-What is your/their role within the team of specialists with regard to treatment/follow-up decisions?

-What are your/their strengths in the follow-up?

-What are your/their limitations in the follow-up?

-The role of the patient in the follow-up:

- **Can you describe the patient’s role in his/her follow-up?**

Prompts:

-What is his/her role in the follow-up?

-How does he/she participate in interprofessional collaboration and the transmission of information between healthcare professionals?

- Do you think that the patient has a role to play in his/her follow-up? If so, which roles and how? And why is that?

- Do you think that the patient has a role to play in interprofessional collaboration?
